# Three Malagasy Cases of Severe Bone Complications Revealing Primary Hyperparathyroidism

**DOI:** 10.1155/crie/9622659

**Published:** 2026-01-20

**Authors:** Zolalaina Andrianadison, Lalao Nomenjanahary Rakotonirina, Oliva Henintsoa Rakotonirainy, Mamonjisoa Olivier Andrianiaina, Mbola Narison Lala Rakotomahefa, Fahafahantsoa Rapelanoro Rabenja

**Affiliations:** ^1^ Department of Pediatric, Joseph Raseta Befelatanana University Hospital, Antananarivo, Madagascar; ^2^ Department of Rheumatology-Dermatology, Joseph Raseta Befelatanana University Hospital, Antananarivo, Madagascar; ^3^ Department of Internal Medicine, Joseph Raseta Befelatanana University Hospital, Antananarivo, Madagascar; ^4^ Department of Rheumatology-Dermatology, Joseph Raseta Befelatanana University Hospital, Faculty of Medicine, University of Antananarivo, Madagascar, univ-antananarivo.mg

**Keywords:** bone deformation, child, pathological fracture, primary hyperparathyroidism, serum calcium

## Abstract

Primary hyperparathyroidism (PHPT) is a metabolic disorder characterized by hypercalcemia with elevated or unsuppressed parathyroid hormone (PTH). It is rare in children but common in adults, particularly in women around the age of 50. In 85% of cases, PHPT is asymptomatic and is diagnosed following hypercalcemia and elevated PTH levels during routine examinations. PHPT occurring before the age of 25 and the normocalcemic phenotype are unusual situations that may delay diagnosis. These nonclassical forms expose patients to severe bone complications that can cause disability. Our objective is to report three clinical cases of PHPT revealed by catastrophic skeletal deformities and pathological fractures.

**Summary**

Primary hyperparathyroidism (PHPT) in its nonclassical forms is often discovered due to severe bone complications, underscoring the importance of systematic medical checkups, particularly for the pediatric population, considering their somatic development. In the presence of bone‐related symptoms in the general population, measurement of serum calcium and parathyroid hormone (PTH) levels before initiating any therapeutic intervention is essential.

## 1. Introduction

Primary hyperparathyroidism (PHPT) is a metabolic disorder characterized by hypercalcemia with elevated or nonsuppressed parathyroid hormone (PTH) levels [1, 2]. It is a common condition among women in their fifties and rare in the pediatric population [3–5]. The prevalence was estimated to range between 17 and 94.6 per 100,000 patient‐years [6]. Sporadic PHPT is caused by a parathyroid adenoma in 85% of cases, multiple parathyroid gland hyperplasia in 15% of cases, and, exceptionally, a malignant form in less than 1% of cases [7, 8]. The incidence of parathyroid carcinoma in adults is increasing. For instance, in Finland, it rose from 1.8 cases per 10,000,000 inhabitants annually between 1970 and 1974 to 10.4 cases between 2010 and 2013 [9]. In children, the prevalence of parathyroid carcinoma is exceedingly rare, with only 14 cases reported up to 2019 [10]. In its contemporary form, asymptomatic PHPT is the most common presentation in contrast to its classical form [6, 11]. Diagnosis is often incidental during routine evaluations due to elevated serum calcium and PTH levels. A calcium‐to‐phosphate ratio equal to or greater than 3.3 has recently emerged as a useful diagnostic indicator [12]. Symptomatic forms reflect the severity of the disease, predominantly affecting the skeletal and renal systems [11, 13]. Bone manifestations of PHPT are almost overlooked in Western countries. However, PHPT in children and normocalcemic variants are rare, with respective prevalences of 0.002–0.005% and 0.1–0.7% [4, 5, 14]. In these atypical forms, diagnosis is often complex and revealed by severe musculoskeletal complications. Our objective is to report, through three clinical cases, severe skeletal deformities and pathological fractures that were indicative of PHPT.

### 1.1. Case 1

A 16‐year‐old adolescent referred for an initial consultation and managed in the rheumatology department for multiple bone deformities. The deformities had appeared over the past year and successively affected his knees, forearms, chest, and spine. He had noted instability while walking, initially painless, which progressively became painful in the hips, necessitating the use of crutches. This instability led to falls from his height, resulting in successive fractures of the left forearm and then the right humerus. He had no personal or family history of endocrine disorders. He had never undergone any clinical investigations prior to this consultation.

**Figure 1:**
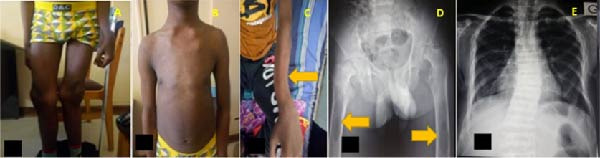
(A) Valgus deformity of the knees, (B) inverted funnel chest deformity, (C) curved deformity of long bones (left forearm), (D) X‐ray of the pelvis and upper third of the femurs showing the presence of brown spots (yellow arrows), and (E): dorsal scoliosis.

He exhibited moderate growth delay (height/age < −2 SD, weight/height < −2 SD). The osteoarticular examination revealed diffuse bone deformities affecting both axial and peripheral skeletons, presenting as valgus knee (Figure 1A), funnel chest (Figure 1B), slight dorsal scoliosis, and a curved deformation of the forearm (Figure 1C). Hip examination showed exquisite tenderness on palpation of the inguinal region, especially on the left, femoroacetabular impingement, and right pelvic tilt. The patient was dehydrated. Cardiovascular examination was normal, and the rest of the clinical examination was unremarkable.

Biological tests revealed corrected hypercalcemia at 2.77 mmol/L, hypophosphatemia at 0.82 mmol/L, calcemia/phosphoremia ratio is 3.37, elevated alkaline phosphatase up to 30 times the normal level, elevated PTH at 1871 pg/mL, and a deficiency in 25‐hydroxyvitamin D3 (25‐(OH)‐D) at 10.8 ng/mL. The complete blood count, C‐reactive protein (CRP), renal function, liver function tests, and serum protein electrophoresis were normal.

Radiographic images showed diffuse bone demineralization with cortical thinning and trabecular bone rarefaction. In the pelvis, brown spots were observed in the upper third of the right femur, and nondisplaced fractures of both femoral heads were noted (Figure 1D). The radiographic image of the dorsal spine showed slight dorsal scoliosis (Figure 1E). The diagnosis of PHPT was made. The patient was lost to follow‐up as imaging for the discovery of the parathyroid tumor was pending.

The patient was treated with a 5 mg infusion of zoledronic acid, vitamin D supplementation, and hydration.

### 1.2. Case 2

**Figure 2:**
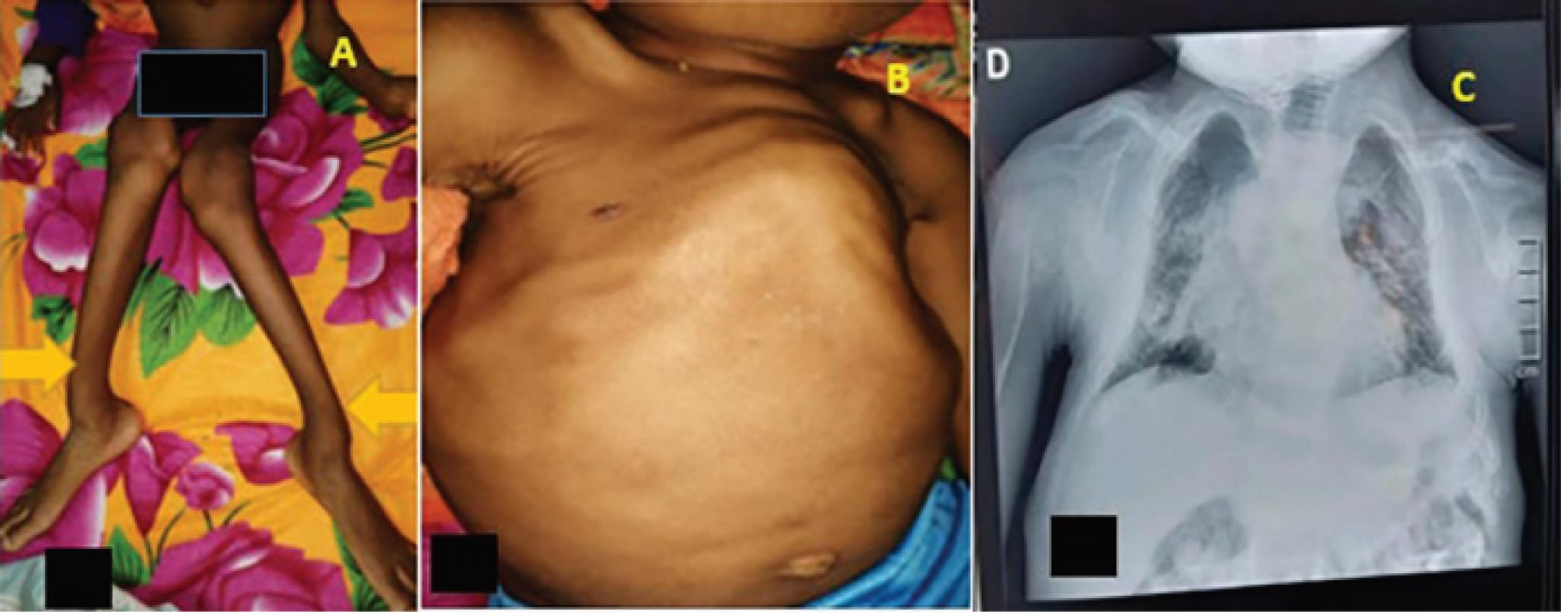
(A) Valgus deformity of the knees and curved deformity of long bones (yellow arrows), (B) inverted funnel chest deformity, and (C) dorsal scoliosis.

A 10‐year‐old boy hospitalized in the pediatrics department for respiratory difficulties and bone deformities affecting the thorax and lower limbs. The condition had been evolving for 2 years with the progressive appearance of painless deformities in the lower limbs and thorax, leading to absolute functional impairment and respiratory difficulties. The clinical examination revealed a child of short stature (height/age < −3 SD) with a disproportionate body. He exhibited diffuse skeletal deformities, including valgus knees (Figure 2A), curved long bones (Figure 2A), bony enlargements at the wrists, ribs, and ankles, an inverted funnel chest (Figure 2B), and dorsal scoliosis with generalized muscle atrophy causing total loss of the ability to stand and sit.

Biological tests showed corrected hypercalcemia at 3.07 mmol/L, phosphatemia at 0.93 mmol/L, calcemia/phosphoremia ratio is 3.3, elevated PTH at 2613 pg/mL, elevated alkaline phosphatase at 3669 IU, and a deficiency in 25‐hydroxyvitamin D3 at 7.1 ng/mL. Renal and hepatic functions were normal.

Radiography revealed diffuse bone demineralization, curved long bones, and dorsal scoliosis (Figure 2C). Cervical ultrasound and cervicothoracic CT scans with and without contrast identified a parathyroid nodule. The diagnosis of PHPT was confirmed.

Symptomatic treatment was initiated, including hydration, a diuretic, a 5 mg dose of zoledronic acid, and vitamin D supplementation. Parathyroid surgery was declined by the family.

### 1.3. Case 3

A 43‐year‐old woman admitted to the rheumatology department for pain in the right lower limb. Four months prior to the consultation, she had undergone surgical removal of a 4 cm bladder stone of unknown nature.

The patient reported diffuse, nonspecific, and disabling mechanical pain in the lower limbs for the past year, which significantly reduced her walking distance to less than 500 m. She also noted a sensation of shortening in the right lower limb. This pain was associated with a progressive decline in general health, characterized by asthenia and a weight loss of 6 kg over the past year.

Physical examination revealed severe malnutrition with a BMI of 11.56 kg/m^2^ (25 kg weight for 147 cm height) and asthenia with a Karnofsky performance score (KPS) of 3. The osteoarticular examination showed valgus knee, a discrepancy in lower limb length with a 1.5 cm shortening on the right, and dorsal scoliosis with right convexity. No palpable masses were found on the rest of the examination.

Biological tests showed normal corrected calcium levels at 2.29 mmol/L, phosphatemia at 0.67mmol/L, calcemia/phosphatemia ratio is 3,4, elevated PTH at 970 pg/L (64 times the normal level), vitamin D deficiency with a serum 25‐(OH)‐D level of 20 ng/mL, normal serum protein electrophoresis, normal complete blood count, negative CRP, ESR at 17 mm, and creatinine at 70 µmol/L.

**Figure 3:**
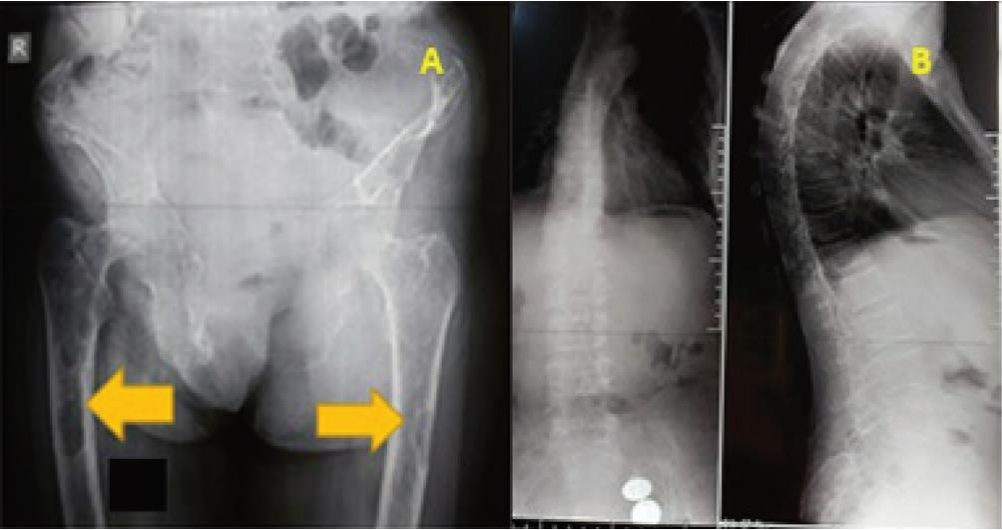
(A) X‐ray of the pelvis and upper third of the femurs showing the presence of brown spots (yellow arrows) and (B) X‐ray of the thoracolumbar spine; staged vertebral compression from T7 to L2 without cortical rupture or posterior wall retraction but with bone rarefaction.

Radiography of the pelvis and femur revealed diffuse bone demineralization and brown tumors in the upper third of both femurs (Figure 3A). Dorsolumbar spine radiography showed staged vertebral collapse from T7 to L2 without cortical rupture or posterior wall retraction but with bone rarefaction (Figure 3B).

Cervical ultrasound revealed hypertrophy of the left lower parathyroid gland.

The patient received a 5 mg infusion of zoledronic acid and vitamin D supplementation. One year later, he underwent surgical removal of a left parathyroid adenoma. Pathological examination identified a parathyroid adenoma with chief cells. The postoperative course was marked by severe asymptomatic hypocalcemia that persisted despite 8 g of injectable calcium supplementation. PTH levels were normal on postoperative Day 4 but increased again after 2 months.

## 2. Discussion

Our report of three clinical cases highlights the clinical and radiological characteristics of bone manifestations in PHPT. The unique aspects of our patients include the description of PHPT in children and adolescents (Cases 1 and 2) and the form mimicking the normocalcemic phenotype (Case 3), which is a recently described phenotype. The limitation of this case series lies in the absence of histopathological evaluation and the loss to follow‐up of patients, which precluded a histological diagnosis of PHPT.

Since the introduction of routine serum calcium measurement in outpatient settings in 1970, the clinical presentation of PHPT has evolved from a symptomatic profile to an asymptomatic one [14, 15]. PHPT is most often asymptomatic and quickly diagnosed in the presence of hypercalcemia and elevated PTH levels [8]. However, PHPT in children and adolescents remains symptomatic, and routine measurement of serum calcium is not a common practice in low‐income countries such as ours [13, 16]. In the pediatric population, hypercalcemia has multiple etiologies, with PHPT accounting for less than 5% of cases of hypercalcemia in children [13]. In the neonatal period, the discovery of severe hypercalcemia accompanied by bone involvement should prompt suspicion of a genetic anomaly due to inactivating mutations of the CASR gene. The diagnosis of neonatal hyperparathyroidism is confirmed by elevated PTH levels, hypocalciuria, and a family history of hyperparathyroidism. In contrast, in children and adolescents, hyperparathyroidism is most often secondary to an adenoma [17]. Our patients correspond to the symptomatic form of PHPT in children and adolescents (Cases 1 and 2), as well as to the form mimicking the normocalcemic phenotype observed in adults (Case 3). No paraclinical investigations, including serum calcium measurement, had been performed in our three patients prior to these consultations.

Bone disease in PHPT is described as osteitis fibrosa cystica. It is characterized by bone pain, fractures, brown tumors, and cysts [18, 19]. The musculoskeletal manifestations of PHPT can vary from simple bone pain and limping to significant skeletal deformities or even cascading pathological fractures. All three patients in our series presented at a stage of very severe bone complications. The diagnostic delay of 1 –2 years can be attributed to ignorance and the lack of calcium‐phosphate evaluation in general medical practice. Due to difficulties in accessing healthcare, disparities exist between countries. In developing nations, according to Yadav et al. [20] the symptomatic form is observed in 79.6% of cases, with musculoskeletal manifestations in 52.9% of cases. In South Africa, the disease is symptomatic in 62.7% of cases [21]. Conversely, in the United States and Canada, the disease is asymptomatic in 95% of cases [22].

Upon initial assessment of our patients, the presence of clinical bone signs led us to order minimum biological tests, including calcium‐phosphate evaluation, renal function, inflammatory markers, alkaline phosphatase, serum protein electrophoresis, intact PTH, and 25‐(OH)‐D levels. In Cases 1 and 2, diagnosis was straightforward due to elevated calcium and serum PTH levels. In Case 3, a normal calcium level but persistently high serum PTH allowed us to establish the diagnosis of PHPT.

Moreover, low 25‐(OH)‐D levels were present in two of our patients (Cases 1 and 2). Vitamin D deficiency is common in PHPT patients. The low 25‐(OH)‐D levels in children, associated with bone deformities and growth retardation, suggest possible rickets, especially in developing countries like ours, where malnutrition remains a public health issue [23, 24]. However, this low level combined with elevated serum PTH, calcium, and alkaline phosphatase levels points directly to PHPT.

Vitamin D deficiency also contributes to significant bone remodeling, particularly in the femoral neck, distal radius, and throughout the body except the lumbar spine. These conditions exacerbate bone manifestations in PHPT. Consequently, routine measurement of 25‐(OH)‐D and careful correction of any deficiency are recommended in PHPT [25–27]. Beyond its diagnostic relevance in PHPT, a calcium‐to‐phosphate ratio greater than 3.3 has also been associated with serum 25‐(OH)‐D levels below 20 μg/L, as well as with the presence of osteitis fibrosa cystica [12], as observed in this case series. Vitamin D deficiency may also reduce the effectiveness of PTH in increasing serum calcium. It could promote parathyroid hyperplasia as well as enlargement of the parathyroid adenoma [28–30]. The third case in this series illustrates this observation.

Radiological imaging is crucial for assessing bone involvement. It reveals classic bone lesions of osteitis fibrosa cystica: a “salt‐and‐pepper” appearance in the skull, bone erosions, phalangeal bone resorption, brown tumors, cysts, and osteoclastomas, which are typical radiological signs of severe hyperparathyroidism [11, 18]. PHPT also causes a reduction in bone mineral density, particularly at cortical sites compared to trabecular sites [11, 18]. This increases the risk of distal long bone fractures and reduces the risk of vertebral fractures [11]. Radiological imaging in these three cases showed diffuse bone demineralization, femoral head fractures, vertebral fractures, and brown tumors.

Biological testing is key for confirming the diagnosis of PHPT. Localization studies using cervical ultrasound and MIBI scintigraphy are essential for guiding surgery. Although surgery is the definitive treatment for PHPT, it is indicated primarily for symptomatic patients and may also be suitable for asymptomatic ones.

Conservative treatment is chosen for patients for whom surgery is inappropriate or in cases of previous surgical failure [13, 16]. Despite the presence of severe bone complications in our cases, only one patient underwent parathyroidectomy. This may be due to the high cost of paraclinical exams and surgery. All three patients received conservative medical treatment, including a 5 mg infusion of zoledronic acid and vitamin D supplementation.

The severe, asymptomatic postoperative hypocalcemia observed in Case 3 was likely attributable to hungry bone syndrome. This syndrome is characterized by a rapid, profound (serum calcium <2.1 mmol/L), and prolonged hypocalcemia (lasting more than 4 days post‐surgery), typically occurring after parathyroidectomy for primary or secondary hyperparathyroidism [31]. It is frequently associated with hypophosphatemia and hypomagnesemia and is exacerbated by a marked decline in PTH levels [32].

## Author Contributions

Andrianadison Zolalaina contributed to the conceptualization, formal analysis, and writing of the original draft, as well as its revision and editing. Rakotoniriana Lalao Nomenjanahary and Rakotonirainy Oliva Henintsoa contributed to the writing of the original draft, as well as its revision and editing. Andriainiana Mamonjisoa Olivier validated and visualized the data. Rakotomahefa Mbola Narison Lala and Rapelanoro Rabenja Fahafahantsoa provided resources and superivison.

## Funding

No funding was received for this study.

## Ethics Statement

The authors thank the patient and the patient’s family who provided written consent for this report. The article omits any personal information that might identify the patient. The names and dates on the chest CT scan have been redacted. The authors have included only the information necessary for scientific understanding.

## Consent

Patient and patient’s family have provided written consent for the publication of this report in accordance with the journal consent policy.

## Conflicts of Interest

The authors declare no conflicts of interest.

## Data Availability

The data that support the findings of this study are available from the corresponding author upon reasonable request.
